# Emergence of reconfigurable wires and spinners via dynamic self-assembly

**DOI:** 10.1038/srep09528

**Published:** 2015-03-26

**Authors:** Gasper Kokot, David Piet, George M. Whitesides, Igor S. Aranson, Alexey Snezhko

**Affiliations:** 1 Complex Matter Department, Jozef Stefan Institute, Jamova 39, 1000 Ljubliana, Slovenia; 2 Materials Science Division, Argonne National Laboratory, 9700 South Cass Avenue, Argonne, IL 60439, USA; 3 Department of Engineering Science and Applied Mathematics Northwestern University 2145 Sheridan Road, Evanston, IL 60208, USA; 4 Department of Chemistry and Chemical Biology, Harvard University, 12 Oxford Street, Cambridge, MA 02138, USA

## Abstract

Dissipative colloidal materials use energy to generate and maintain structural complexity. The energy injection rate, and properties of the environment are important control parameters that influence the outcome of dynamic self-assembly. Here we demonstrate that dispersions of magnetic microparticles confined at the air-liquid interface, and energized by a uniaxial in-plane alternating magnetic field, self-assemble into a variety of structures that range from pulsating clusters and single-particle-thick wires to dynamic arrays of spinners (self-assembled short chains) rotating in either direction. The spinners emerge via spontaneous breaking of the uniaxial symmetry of the energizing magnetic field. Demonstration of the formation and disaggregation of particle assemblies suggests strategies to form new meso-scale structures with the potential to perform functions such as mixing and sensing.

The use of self-assembly to generate functional structures that have sizes or properties not otherwise accessible has been a largely unrealized objective of materials science for decades. Examples of partial successes include nanoparticles and aggregates in biosensing^1^,^2^, ferrofluids and magneto-rheological liquids for clutches and bearings, and optical colloids^3^,^4^. Most of these systems are at equilibrium or steady state.

Out-of-equilibrium (active) colloidal suspensions^5^,^6^,^7^,^8^,^9^,^10^,^11^,^12^,^13^,^14^,^15^,^16^,^17^,^18^,^19^ are much more challenging. Model colloids have helped to understand the principles that guide dynamic self-assembly: those systems offer a variety of properties due to their high structural controllability, and the range of possible interactions among them^20^,^21^. External electro-magnetic fields^22^,^23^,^24^,^25^, UV radiation^26^ or chemical reactions^27^ have been used to supply energy for dynamic colloidal self-assembly. The resulting dynamic structures^8^,^9^,^19^,^28^,^29^ are usually unique, and not accessible under equilibrium conditions. Complex collective motion and hierarchical ordering in these out-of-equilibrium systems reflect the balance between many types of interactions among particles, ranging from short-range steric to long-range hydrodynamic and electromagnetic ones. Here we report on the emergent dynamics, and resulting self-assembly of suspensions of ferromagnetic microparticles confined at a liquid-air interface, and energized by a uniform in-plane uniaxial alternating magnetic field (see Methods). Reported self-assembled structures range from dynamic wires and clusters to spinners. The alternating magnetic field maintains the system out of equilibrium, and controls both structural morphology and time-dependent behavior of the structures.

## Results

**Figure 1:**
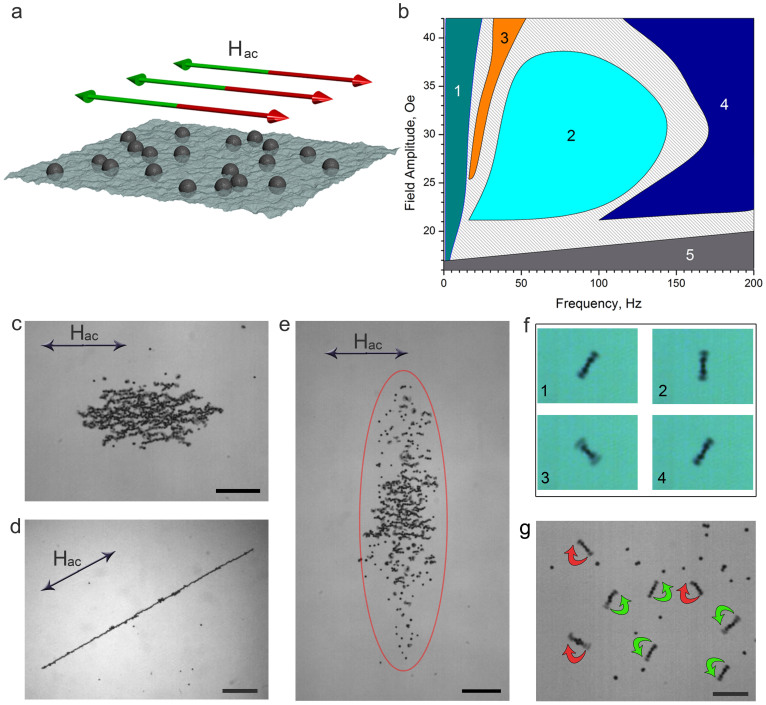
Dynamic self-assembled structures at the air-liquid interface. (a) Schematics of the experiment. A uni-axial alternating magnetic field is applied parallel to the air-liquid interface. (b) Phase diagram illustrating different states of magnetic dispersion (~10^2^ −10^3^ particles) versus the amplitude and frequency of the applied magnetic field. Region 1 depicts pulsating clusters. Region 2 corresponds to a gas of spinners. Region 3 delineates phase boundaries of the perpendicular cloud phase. Region 4 corresponds to dynamic wires. Particles form dense static clusters in Region 5. Regions marked with diagonal lines pattern indicate a wide hysteretic domain where neighboring structures co-exist. (c) Loose pulsating cluster observed at 20 Hz, 27 Oe magnetic field. Scale bar is 2 mm. (d) Dynamic particle-thick wire formed at 180 Hz, 40 Oe magnetic field. Scale bar is 2 mm. (e) Snapshot of a perpendicular cloud consisting of continuously rearranging short chains. The cloud is extended perpendicular to the axis of the applied magnetic field (40 Hz, 40 Oe) in striking contrast to the cluster phase or dynamic wires. Scale bar is 2 mm. (f) Dynamics of a single spinner within one period of the driving alternating magnetic field (29 Oe, 50 Hz). Length of the spinner is 460 μm. (g) Spinner phase formed at 50 Hz, 29 Oe magnetic field. Short chains of particles rotate in either direction with the frequency of the applied magnetic field. Clock- and anti-clock-wise spinners are illustrated by arrows. Scale bar is 1 mm.

Surface tension confines the ferromagnetic microparticles (~90 μm) with intrinsically pinned magnetic moments (see Methods) to the surface of the water-air interface. An alternating magnetic field applied parallel to the interface (Fig. 1a) exerts torque on them. The torque is dissipated locally in the liquid and generates hydrodynamic flows around each particle. Consequently, the particles interact by two temporally related, but physically distinct types of forces: magnetic (dipole-dipole interactions) and hydrodynamic. Dynamic self-assembly reflects the interplay between magnetic interactions and hydrodynamic flows. The relative contributions of these two primary interactions are modulated by the parameters of the energizing alternating magnetic field. As a result, the system exhibits a remarkable diversity of quasi-stable dynamic states. The phase diagram in Fig. 1b delineates these various self-assembled phases as a function of the frequency and amplitude of the applied magnetic field. All the states are dynamic, and exist only while energy is supplied by the *a.c.* magnetic field. Fig. 1c-g illustrates major distinctive phases.

Loose clusters extended along the *a.c.* magnetic field are formed at low frequencies. The clusters exhibit periodic changes in shape (pulsations): over time the cluster extends and contracts with a fraction of the driving magnetic field frequency (see Supplementary Video 1).

At elevated frequencies of the applied field, the cluster transforms into a cloud of continuously rearranging short chains (Fig. 1e). In striking contrast with a loose cluster, the cloud switches the axis of elongation and extends perpendicular to the *a.c.* magnetic field. Further increase in the frequency of the applied magnetic field yields a new dynamic phase: spinners (see Fig. 1f,g). In this phase the particles self-assemble into short chains, and rotate in the plain of the air-water interface at the frequency of the applied *a.c*. magnetic field. This rotation creates strong in-plane hydrodynamic flows. The spinners exhibit complex dynamic behavior: they move across the surface, collide, disintegrate, and re-assemble (see Supplementary Video 2 and 3). The multi-spinner state (gas) has no apparent preferred direction and covers the entire area of the container uniformly. At higher frequencies of the applied field the spinners give way to dynamic wires (see Fig. 1d). All phases form and disassemble reversibly (see Supplementary Video 4), and their behavior is controlled by the parameters of the applied *a.c*. magnetic field.

### Dynamic self-assembled wires

**Figure 2:**
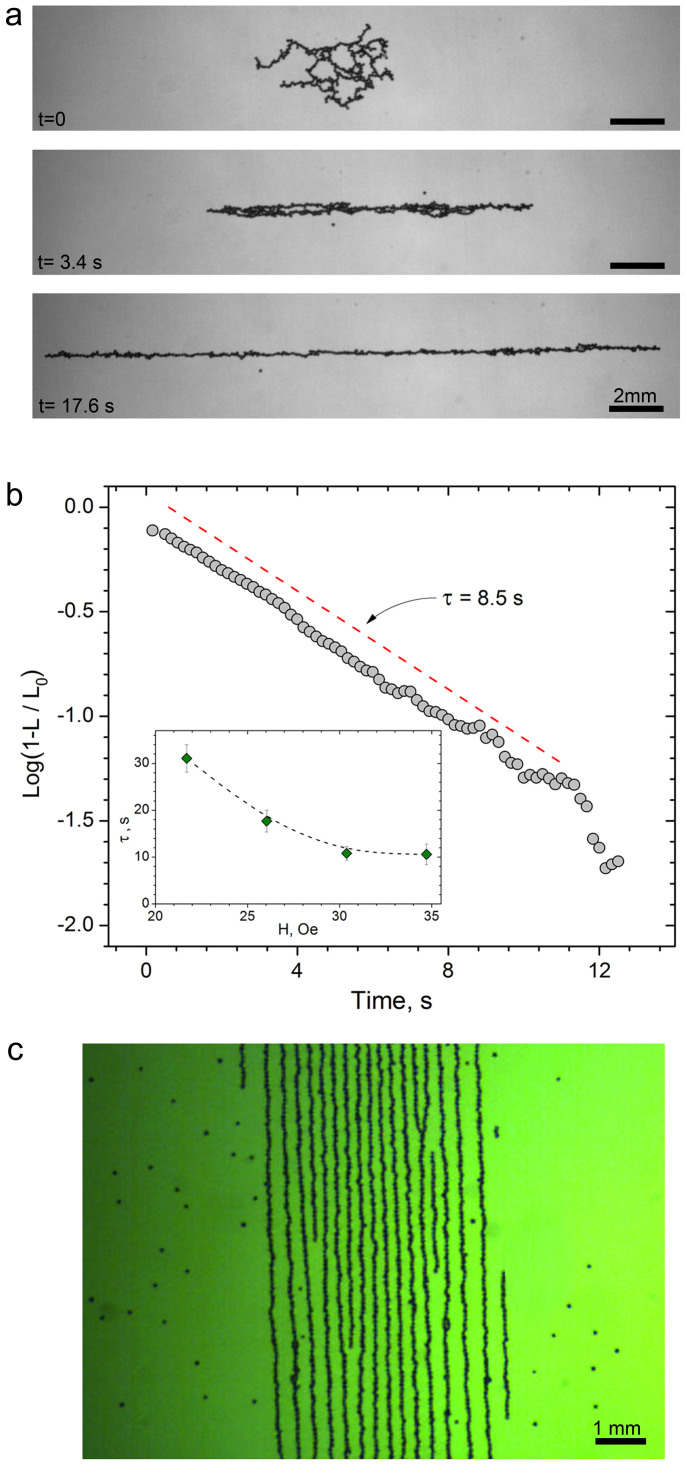
Self-assembly of dynamic magnetic wires. (a) Consecutive formation of the dynamic wire from a compact cluster at 36 Oe, 240 Hz in-plane magnetic field. Formation of a dynamic wire proceeds along the axis of the applied field. (b) Time evolution of the rescaled dynamic wire length. The wire was assembled at 35 Oe, 260 Hz applied field. Wire formation proceeds through deformation of the cluster of particles rather than aggregation phenomenon. Insert: dependence of the wire formation characteristic time on the amplitude of in-plane magnetic field. The frequency of excitations was fixed at 260 Hz. (c) Parallel arrays of particle-thick dynamic wires assembled with 54 Oe and 200 Hz in-plane field. Additional 20 Oe static magnetic field was applied perpendicular to the interface to stabilize the pattern.

The formation of dynamic wires proceeds through an elongation of the initial cluster of particles along the axis of the applied magnetic field (see Fig. 2a). During its growth, the wire shows imperfections (e.g. kinks and perforations). These are eventually eliminated by the rearrangements of near-by particles. The extension of a wire continues until it becomes one-particle thick (see Supplementary Video 5). Since all dynamic phases are reversible and controlled by the parameters of the applied *a.c.* field, the formation of a wire is also possible from the initial spinner phase by adjustment of the a.c. field frequency. In this case, growth of the wire proceeds by successive attachments of individual particles and merging of linear chain fragments (see Supplementary Video 6).

To characterize the dynamics of growth of the wires, we monitored the evolution of the length of a wire as a function of the frequency and amplitude of the driving magnetic field within a parameter space of a wire phase. Fig. 2a shows the stages of formation of a typical wire. Initially all particles aggregate in a static compact cluster due to ferromagnetic interactions among particles: strong magnetic dipole-dipole inter-particle interactions exist even in the absence of the external magnetic field. Upon application of the alternating magnetic field, the static cluster transforms into a thick non-uniform bundle aligned along the field axis and continues to extend until it reaches a single-particle thickness. This process contrasts with a common mode of aggregation^30^ where growth proceeds through a succession of attachments (aggregation) of individual particles from the surrounding surface. The dynamics of formation of wires can be approximated by the following dependence, 
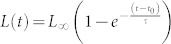
. Here *L*_∞_ is the established asymptotic length of the wire at time *t* → ∞; *t_0_* is the time when the *a.c*. field was applied; and τ is a characteristic time for wire growth. The saturation of growth is caused by the depletion of particles in the vicinity of the wire. Fig. 2b shows a typical evolution of the length of the wire with time. From this type of analysis, we have extracted the characteristic growth times, τ, for different parameters of the applied magnetic field. Increases in the magnitude of the applied field significantly decrease the characteristic formation time (see inset to Fig. 2b). In contrast, variations in frequency of the applied field (within the boundaries of the dynamic wire phase) do not produce detectable changes in the characteristic formation time.

Reversible assembly of straight wires from ferromagnetic particles is a challenging task. Under equilibrium conditions, a static magnetic field will produce only a deformed compact cluster of ferromagnetic particles. Out-of-equilibrium dynamics enables assembly of a more complex wire pattern in this system: parallel arrays of single-particle-thick wires (Fig. 2c). For this case, an additional small static (*d.c*.) magnetic field (20 Oe) applied perpendicular to the interface was added to stabilize the pattern. The spacing between wires in the array is determined by the magnitude of the *d.c.* field and the meniscus: the spacing is slightly smaller in the middle of the array. Upon removal of the stabilizing field the array of wires evolves towards formation of a single wire by successive coalescence of separate wires (seen as “Y”-shaped defects in Fig. 2c).

### Spinners and spontaneous symmetry breaking

Surprisingly, in a certain range of parameters for the applied field (region 2 in Fig. 1b) a striking transformation takes place: the uniaxial symmetry of the field breaks spontaneously, and an ensemble of spinners rotating both clockwise and counterclockwise emerges, see Fig. 1f,g and Supplementary Video 3. Dynamic spinners are composed of short self-assembled chains of micro-particles ferromagnetically ordered due to the prevailing magnetic dipole-dipole inter-particle interactions. Spinners rotate clockwise or counterclockwise with the frequency of the applied field. The onset of spinning is related to the following mechanism. Individual ferromagnetic particles aggregate into short chains with the resulting magnetic moment oriented along the chains. The applied *a.c*. magnetic field forces the chain to align with the field orientation, resulting in periodic (with the field frequency) reversals of the direction. If the chain's rotational inertia is large compared to the viscous drag, the chain will preferably rotate in one of the directions. Since for the case of uniaxial magnetic field the clock/counterclockwise senses of rotation are equally probable, the initial direction is selected by a variety of factors, like interactions with neighboring particles and flows.

The rotating chains exert viscous torques on the liquid which trigger strong long-range hydrodynamic vortical flows at the interface. In the bulk, the spinners create swirling flow, which decays roughly as the square of the depth. Since our experiments are performed in deep containers (the ration of the depth of the container to the length of the spinners is of the order of 100), the effects of the container's bottom on the vortical flow at the interface are negligible.

**Figure 3:**
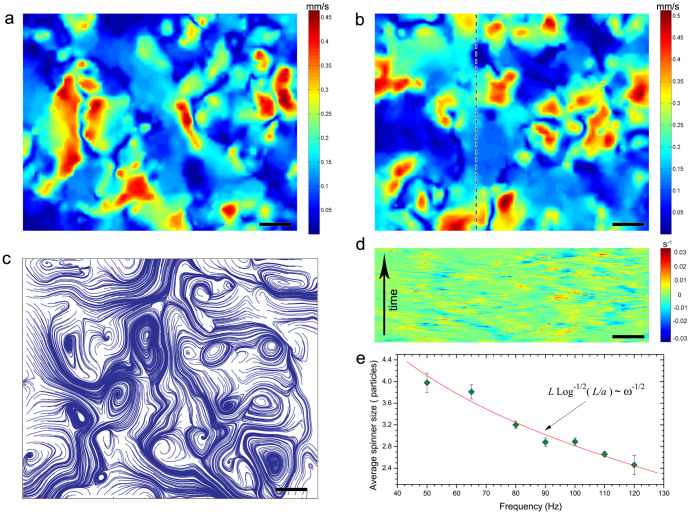
Hydrodynamic surface flow generated by self-assembled dynamic spinners. (a),(b) Magnitude of the hydrodynamic flow velocity generated by spinners at the air-liquid interface at time *t_1_ = 6.88* *s* and *t_2_ = 18.28* *s* respectively. The system was energized for 4 minutes before the start of the image acquisition in a fully developed spinner phase. Flow velocity fields were obtained by a particle-image velocimetry (PIV), see Methods. The in-plane applied alternating magnetic field is 29 Oe, 70 Hz. Scale bar is 2 mm. (c) A typical streamline pattern of hydrodynamic surface flows. The data was collected for spinners generated at 29 Oe, 70 Hz applied field. Spinners produce a complex time dependent vorticity distribution at the liquid interface. Scale bar is 2 mm. (d) Space-time diagram of the flow vorticity field along a fixed slice of the system shown as a dashed line in (b). The time interval spans 40 s and includes 200 slices. Spinners, advected by the flow, create rapidly changing flow velocity fields at the liquid interface. Domains of fast/slow flow appear as short red and blue dashes in the space-time plot. Slope of red/blue dashed lines characterizes advection velocity of the spinners. Scale bar is 2 mm. (e) Average spinner size as a function of frequency of the applied in-plane magnetic field. Solid line is a fit to the *L log^−1/2^ (L/a) ~* *ω^−1/2^* law following from the balance between magnetic and hydrodynamics torques. Here *L* is a spinner length, *a* is a particle diameter. The data was collected for 29 Oe amplitude of the applied magnetic field.

Spinners move (seemingly randomly) due to magnetic interactions and the flows generated by other spinners. Their collective motion creates an overall gas-like appearance of the phase (see Supplementary Video 2). The structure of each spinner is not fixed. Spinners collide, disintegrate and reassemble, and thus create complex time-dependent hydrodynamic patterns at the interface. The hydrodynamic surface flows generated by spinners have been characterized by particle-image velocimetry (Methods). Fig. 3a,b shows snapshots of the velocity field for the same system at two different times.

Fig. 3c shows streamlines of a typical velocity field generated by the spinners at the air-liquid interface. The pattern of surface flow exhibits complex spatio-temporal dynamics, manifested by abrupt local bursts and subsequent cessation of activity (Fig. 3d). The characteristic size reached by the spinners depends on the frequency of the applied *a.c.* field. An increase in the frequency of the field shortens the length of the spinning chains. The length is determined by a balance between magnetic and viscous torques acting on a chain at the liquid interface. Balancing the torques, we find (in the slender body approximation)^31^ that 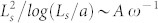. Here, *L_s_* is a characteristic length of a spinner, *a* is a particle diameter, and *A* is a factor that depends on magnetic moment of the particles, their radius, the amplitude of the applied magnetic field and the viscosity of the liquid. The expression provides an adequate description of the experimentally observed behavior (Fig. 3e).

### Simulations

**Figure 4:**
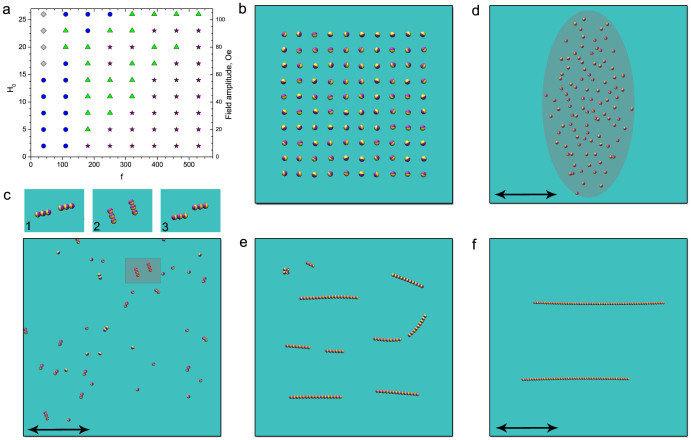
Simulations of dynamic phases. (a) Phase diagram of the dynamic states versus the magnitude and frequency of the alternating magnetic field as obtained from the simulations. Stars depict dynamic wires. Circles correspond to a gas of spinners. Diamonds delineate the perpendicular cloud phase. Triangles correspond to the simulation results where a mixture of different dynamic structures has been observed. Field amplitude *H_0_* is rescaled by *μ/(4πa*^*3*^*)*, where *μ* is a magnetic moment per particle. Frequency is scaled by 1 Hz. Right axis shows recalculated values of the field amplitude for the particles used in the experiments. (b) Starting configuration of the magnetic suspension in the simulations: particles with initially random orientations are distributed on a perturbed square lattice. Each particle is shown as a sphere with a darker hemisphere corresponding to a magnetic “north pole”. (c) Spinners phase as obtained from simulations. Top sequence of images illustrates rotation of two neighboring spinners over a half-period of the applied magnetic field. Arrows depict the axis of the applied magnetic field. See Supplementary Video 7. (d) Snapshot of a perpendicular cloud phase as obtained from the simulations. The cloud is extended perpendicular to the axis of the applied magnetic field in an agreement with the experimental observations (see Fig. 1e). (e), (f) Formation of dynamic wires. Self-assembly proceeds through a formation and subsequent merging and reorientation of short chain segments. Snapshots of a system after 1700 (e) and 41700 (f) periods of the applied field from the moment the field was turned on. See Supplementary Video 8.

We numerically solved the equations of motion for *N* particles on a plane. Particles are energized by an in-plane uni-axial magnetic field, and interact due to their magnetic dipole moments, self-induced hydrodynamic flows, and steric repulsion. In simulations, the length was scaled on the particle size, time was scaled by 1/*f*, where *f* is the frequency of the applied magnetic field, and field amplitude was scaled by *μ*/(4*πa*^3^), where *μ* is a magnetic moment per particle. The parameters in the equations of motion were calculated from actual properties of the particles and fluid (size, magnetic susceptibility, viscosity). See Methods for the details of the model and simulations. The simulations successfully reproduced the morphology of experimental phase diagram (Fig. 4a) and captured most of the observed phases (see Fig. 4).

### Synchronization of spinners

Strong hydrodynamic and magnetic couplings between the spinners hint at possible synchronization (perhaps similar to synchronization of applause after musical performances^32^, or synchronization of weakly coupled pendulum clocks^33^). While clock/counterclockwise spinners are equally probable, synchronization can lead to the emergence of phases with a dominant direction of rotation.

**Figure 5:**
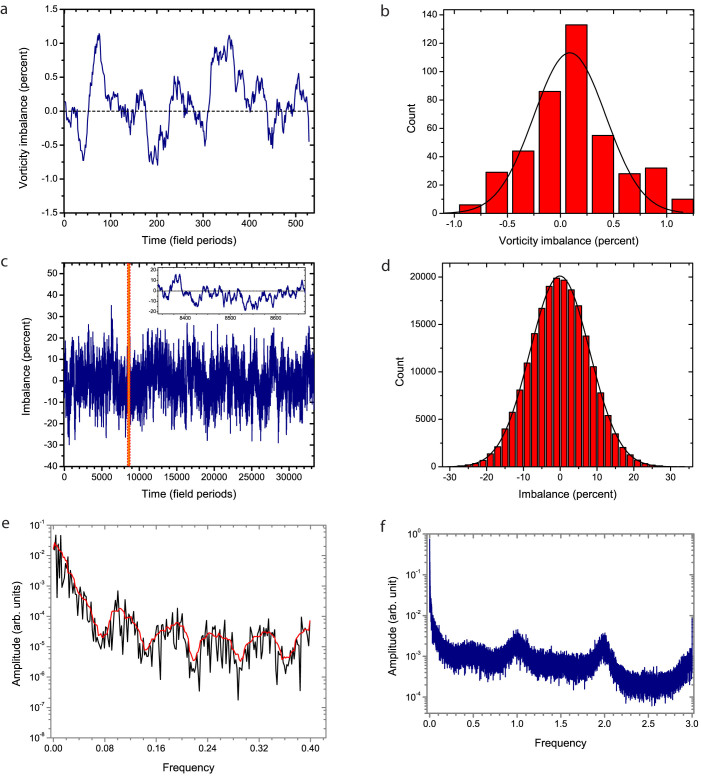
Spontaneous rotational symmetry breaking in spinners phase. (a),(b) Imbalance of positive/negative vorticity generated by clockwise versus counterclockwise spinners at the air-liquid interface. The spinners phase was dynamically self-assembled at 29 Oe, 50 Hz in-plane alternating magnetic field. Vorticity imbalance was calculated as an integral of the vorticity field over the experimental surface window. Each instance of the data contained information about 10^2^ spinners within the experimental image. While the ensemble of spinners constantly develops spontaneous fluctuations in the number of spinners with different sense of rotation demonstrated in (a), the overall long time trend shows no synchronization as illustrated in the histogram of the imbalance events (b). Solid line is a fit to a Gaussian function. (c) Imbalance in number of spinners with different sense of rotation as a function of time obtained from simulations (see Methods). Each time step corresponds to 1/6 of the excitation period of the powering field. The simulation data has been collected over much longer time frame compared to the experiment (two orders of magnitude difference). The inset shows the behavior of the order parameter in the time window comparable to the experimental one shown in (a). The results are in good qualitative agreement. (d) Histogram of the order parameter values as obtained from the simulation data shown in (c). Solid curve is a fit to a Gaussian function. The results indicate an absence of synchronization between spinners. (e), (f) Fourier spectra of the spinners order parameter obtained for the experimental data (e) and simulations (f). The same data as in (a) and (c) are used for analysis. Frequency is in units of inverse field periods. Solid curve in (e) is a running average over neighboring 5 data points to reduce the noise. The clear evidence of peaks in the spectra suggests a deterministic nature of the order parameter fluctuations.

To quantify this effect we introduce an order parameter *I* related to the imbalance of spinners with different sense of rotation: *I* = (*N*_+_ − *N*_−_)/(*N*_+_ + *N*_−_). Here *N_+_* and *N_-_* are the number of clockwise and counterclockwise spinners respectively. The emergence of complete global synchronization of the sense of rotation of spinners rotation would correspond to the order parameter values *I* = ±1; the completely random non-synchronized state would correspond to *I* = 0. The results of experimental and computational studies are illustrated in Fig. 5. Smaller number of particles in a simulation window (see Methods) than those used in experiments expectedly produce smaller amount of spinners per simulation frame (~10). Consequently, asymmetry in the number of spinners with different rotational direction results in a high magnitude of imbalance compared to the larger experimental system (~10^2^ spinners).

While both experiment and simulation revealed significant fluctuations in the order parameter (see Fig. 5a,c), no global synchronization was detected. Analysis of the fluctuation of the order parameter resulted in Gaussian distribution peaking at zero, and suggested no long-lived synchronization events (see Fig. 5d). To better understand the collective dynamics of spinners, we performed a Fourier analysis of the fluctuations in the order parameter. The results are shown in Fig. 5e,f. The spectra reveal pronounced peaks at certain characteristic frequencies (Fig. 5e), suggesting deterministic origin of the seemingly random order parameter fluctuations. Simulations reproduced similar phenomenon (Fig. 5f).

The origin of spontaneous symmetry breaking and rotation is associated with the non-negligible inertia of the colloidal particles and the fluid their motion entrains. Consider for simplicity an individual particle in a uniaxial field *H* = *H*_0_sin(*ωt*). The evolution of a particle's orientation *ϕ* is described by the balance of viscous and magnetic torques and the particle's inertia,
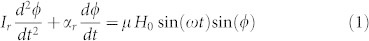
where *I_r_*, *α_r_*, *μ* are the moment of inertia (including the inertia of the entrained liquid) of the particle, the rotational drag, and the magnetic moment. The right part of Eq (1) can be decomposed into clockwise and counter-clockwise rotating waves
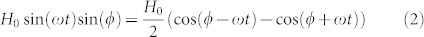
This equation is well-known in the context of deterministic chaos^34^. If we neglect one of the waves in Eq (2), then a fixed point of this equation will correspond to a steady rotation of the particle with frequency ω. The rotation is possible as long as the viscous friction is below a certain threshold, *α_r_ω* < *μ* *H*_0_/2. However, increasing the magnitude of the field *H*_0_ results in an interaction with a counter-rotating wave. It eventually leads to the rotation reversal and the onset of the overall chaotic behavior. The so-called “resonance overlap criterion”^35^ for the onset of chaotic behavior is given approximately by
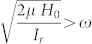


Typical trajectories of Eq (1) are characterized by chaotic reversal of direction of rotation with well-pronounced peaks in the spectrum at the fractions of the driving frequency ω. Corresponding spectral peaks are captured in both experimental data and simulations (see Fig. 5e,f). Quantitative differences between experimental and simulation spectra originate from two major sources: particle size inhomogeneity in the experiment (75–100 μm) and approximations of the model used in the simulations (see Methods).

In conclusion, we have demonstrated that a dispersion of ferromagnetic microparticles, confined at the air-liquid interface and energized by a uniaxial in-plane alternating magnetic field, form a rich variety of nontrivial, dynamically self-assembled states, ranging from pulsating clusters and tunable, single-particle-thick wires, to self-assembled spinners. The morphology and complex collective behavior of these out-of-equilibrium self-assembled structures could be tuned by the parameters of the applied magnetic field. Our study did not reveal global synchronization in the gas of spinners. However, the propensity towards synchronization can be enhanced by the use of bi-axial fields^8^,^11^, e.g. chiral rotational field that will break the clock/anticlockwise rotational symmetry. In this case, one sense of rotation will be more preferred than the opposite.

Our work provides new insights into the engineering of tunable complex dynamic architectures by the means of out-of-equilibrium self-assembly. All reported dynamic structures are fully reversible. Possible applications might include design of wire networks via dynamic self-assembly with a topology controlled by a magnetic field, e.g. for flexible electronics and microrobotics, or non-contact mixing at the interface. Another intriguing extension of our work would be a dynamic self-assembly of ferromagnetic particles suspended in the bulk of the fluid rather than at the interface. This technique, complimentary to self-assembly of diamagnetic particles via magnetic levitation^37^,^38^, has the potential to lead to a variety of novel, three-dimensional, dynamic structures.

## Methods

Ferromagnetically ordered nickel spherical particles (Alfa Aesar Company) with an average diameter of ~90 μm (75–100 μm uniform size distribution) were used in the experiments. Magnetic saturation moment per particle is ~200 μemu at 4 kOe field. Because of intrinsic defects in particles their magnetic moments are strongly pinned, and the particles behave as magnetically “hard” microspheres. The presence of “pinned” magnetic moments in the particles is important prerequisite for observation of reported phenomena. Similar dynamic self-assembly has been observed with smaller particles down to ~30 μm. However, since the spinner phase exists due to inertia of the particles, we anticipate that this phase will disappear for too small particles (below ~10 μm).

Particles (about 10^3^ particles) were dispersed at the interface between deionized water and air. The suspension was energized by a uniform uni-axial alternating magnetic field, *H*_ac_ = *H*_0_ sin(2π*ft*), applied parallel (in-plane) to the interface. The amplitude of the *ac* magnetic field, *H*_0_, was in the range of 10–60 Oe and the frequency, *f*, was in the range of 5–300 Hz. The alternating in-plane magnetic field was created by a pair of custom-made precision Helmholtz coils.

### Optical microscopy and particle image velocimetry

A glass container (5 cm in diameter) containing the particles was mounted on a microscope stage (Leica MZ9.5). The trajectories of the particles were monitored by a fast CCD camera (RedLake MotionPro). The particle image velocimetry (PIV) was performed at the water-air interface. Nonmagnetic tracer particles (copper 10–20 μm spheres) were added at the water interface and were supported by surface tension. We recorded image sequences (1280 × 1024 resolution) at a frame-rate of 400 fps. Image and data analysis of the time sequences were carried out using ImageJ, MatPIV and custom scripts.

### Simulations

To determine positions **r**_i_ and orientations ϕ_i_ of particles with respect to the fixed direction we numerically solved Newton's equations of motion
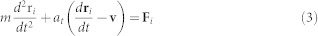

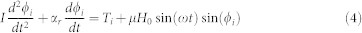
where *m* is the mass of particle, *μ* is its magnetic moment, *I* is moment of inertia, and *α_t,r_* are, respectively, translation and rotation drag coefficients, **F**_i_ and *T*_i_ are forces and torques due to magnetic dipole-dipole interaction and hard-core repulsions (see for detail Refs. 18,36). Hydrodynamic interactions between the particles are described by the advection with velocity **v**, where **v** is the hydrodynamic velocity created due to rotation of all particles (in the Stokes limit, a rotating particle produces azimuthal flow with the velocity 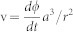, where *a* is the radius of the particle and *r* is the distance from the center). For simplicity we neglected the secondary flow generated by a non-steady rotating particle and realignment of the particles in shear flow. The last term in Eq. (4) describes the torque from the applied magnetic field *H* = *H*_0_ sin(ωt) on a permanent magnetic dipole.

The algorithm was implemented on a GPU card. 100–200 particles were simulated over more than 20,000 periods of the applied field at different parameters of the driving magnetic field.

## Author Contributions

A.S. and G.K designed and carried out the experiments; D.P. and I.S.A developed the model and performed simulations; A.S., I.S.A. and G.M.W. analyzed obtained experimental data and wrote the manuscript.

## Supplementary Material

Supplementary Information Supplementary Information

Supplementary Information Supplementary Video 1

Supplementary Information Supplementary Video 2

Supplementary Information Supplementary Video 3

Supplementary Information Supplementary Video 4

Supplementary Information Supplementary Video 5

Supplementary Information Supplementary Video 6

Supplementary Information Supplementary Video 7

Supplementary Information Supplementary Video 8
