# Supplementary material for: Emergence of reconfigurable wires and spinners via dynamic self-assembly
Source: Sci Rep. 2015 Mar 26;5:9528. doi: 10.1038/srep09528 (PMC4374141; doi:10.1038/srep09528)
Supplement: Supplementary Information [file srep09528-s1.pdf]

Emergence of reconfigurable wires and spinners via dynamic self-assembly

Gasper Kokot[1], David Piet[2,3], George M. Whitesides[4], Igor S. Aranson[2,3], Alexey Snezhko[2*]

Supplementary Video 1. Loose cluster generated at 20 Hz, 28 Oe of the driving in-plane magnetic field.

Supplementary Video 2. Gas-like appearance of the spinner phase. 70 Hz, 28 Oe driving in-plane magnetic field.

Supplementary Video 3. Detailed dynamics of spinners. 80 Hz, 30 Oe driving in-plane field. Playback is 10 times slower than the real time.

Supplementary Video 4. Dynamic self-assembled wire. Frequency sweep 20Hz-300Hz-20 Hz at a rate about 30Hz per 10 sec . Amplitude of the driving in-plane magnetic field is 40 Oe. Playback is 1.2 times faster than the real time.

Supplementary Video 5. Dynamic self-assembled wire. The wire is formed at 200 Hz, 40 Oe driving in-plane magnetic field.

Supplementary Video 6. Dynamic self-assembled wire. The wire is formed at 300 Hz, 40 Oe driving in-plane magnetic field from initial spinner phase (60Hz).

Supplementary Video 7. Simulations results: the spinner phase.

Supplementary Video 8. Simulations results: formation of the dynamic self-assembled wires.
